# Vital Economy or How to Conserve Your Strength

**Published:** 1910-06

**Authors:** 


**Vital Economy or How to Conserve Your Strength.** By **John H. Clarke,** M.D. 12 mo, pp. 119. New York: A. Wessels. 1909. (Cloth, 50 cents.)

The author has written this book for the benefit of those persons whose vitality is only sufficient to enable them to get through their work by exercising care and due economy; or, as Dr. Clarke says in his preface, for those who are "below par." He advises moderation in exercise. The not too frequent use of baths and the avoiding of undue excitement from any cause, which is likely to bring about what he terms "nerve wear" or loss of vital energy. The book is well worth perusal. J. A. R.

